# Supplementary material for: Consistency, completeness and external validity of ethnicity recording in NHS primary care records: a cohort study in 25 million patients’ records at source using OpenSAFELY
Source: BMC Med. 2024 Jul 10;22:288. doi: 10.1186/s12916-024-03499-5 (PMC11234682; doi:10.1186/s12916-024-03499-5)
Supplement: Supplementary file 1 — Additional file 1: Fig. S1. Bar plot showing the proportion of 2021 Census and TPP populations (amended to 2021 grouping) per ethnicity grouped into 5 groups (excluding those without a recorded ethnicity). Annotated with percentage point difference between 2021 Census and TPP populations. Fig S2. Bar plot showing the proportion of 2021 Census and TPP populations (amended to 2021 grouping) per ethnicity grouped into 5 groups per NUTS − 1 region (excluding those without a recorded ethnicity). Annotated with percentage point difference between 2021 Census and TPP populations. Fig. S3. Recording of ethnicity over time for latest and first recorded ethnicity. Unknown dates of recording may be stored as ‘1900 − 01 − 01’. Table S1. Count of patients with a recorded ethnicity in OpenSAFELY-TPP (proportion of registered TPP population) by clinical and demographic subgroups. All counts are rounded to the nearest 5. Table S2. Count of patients with a recorded ethnicity in OpenSAFELY TPP by ethnicity group (proportion of registered TPP population) and clinical and demographic subgroups. All counts are rounded to the nearest 5. Table S3. Count of patients with a recorded ethnicity in OpenSAFELY TPP by ethnicity group (proportion of registered TPP population) and clinical and demographic subgroups. All counts are rounded to the nearest 5. Table S4. Count of patients’ most frequently recorded ethnicity (proportion of latest ethnicity). Table S6. Count of patients with a recorded ethnicity in Secondary Care by ethnicity group excluding Unknown ethnicites (proportion of Primary Care population). All counts are rounded to the nearest 5. Table S7. Count of patients with a recorded ethnicity in OpenSAFELY TPP by ethnicity group (proportion of registered TPP population) and 2021 ONS Census counts [amended to 2001 grouping] (proportion of 2021 ONS Census population). All counts are rounded to the nearest 5. Table S8. Count of patients with a recorded ethnicity in OpenSAFELY TPP [amended [file 12916_2024_3499_MOESM1_ESM.pdf]

Figure 1: Barplot showing the proportion of 2021 Census and TPP populations (amended to 2021 grouping) per ethnicity grouped into 5 groups (excluding those without a recorded ethnicity). Annotated with percentage point difference between 2021 Census and TPP populations.

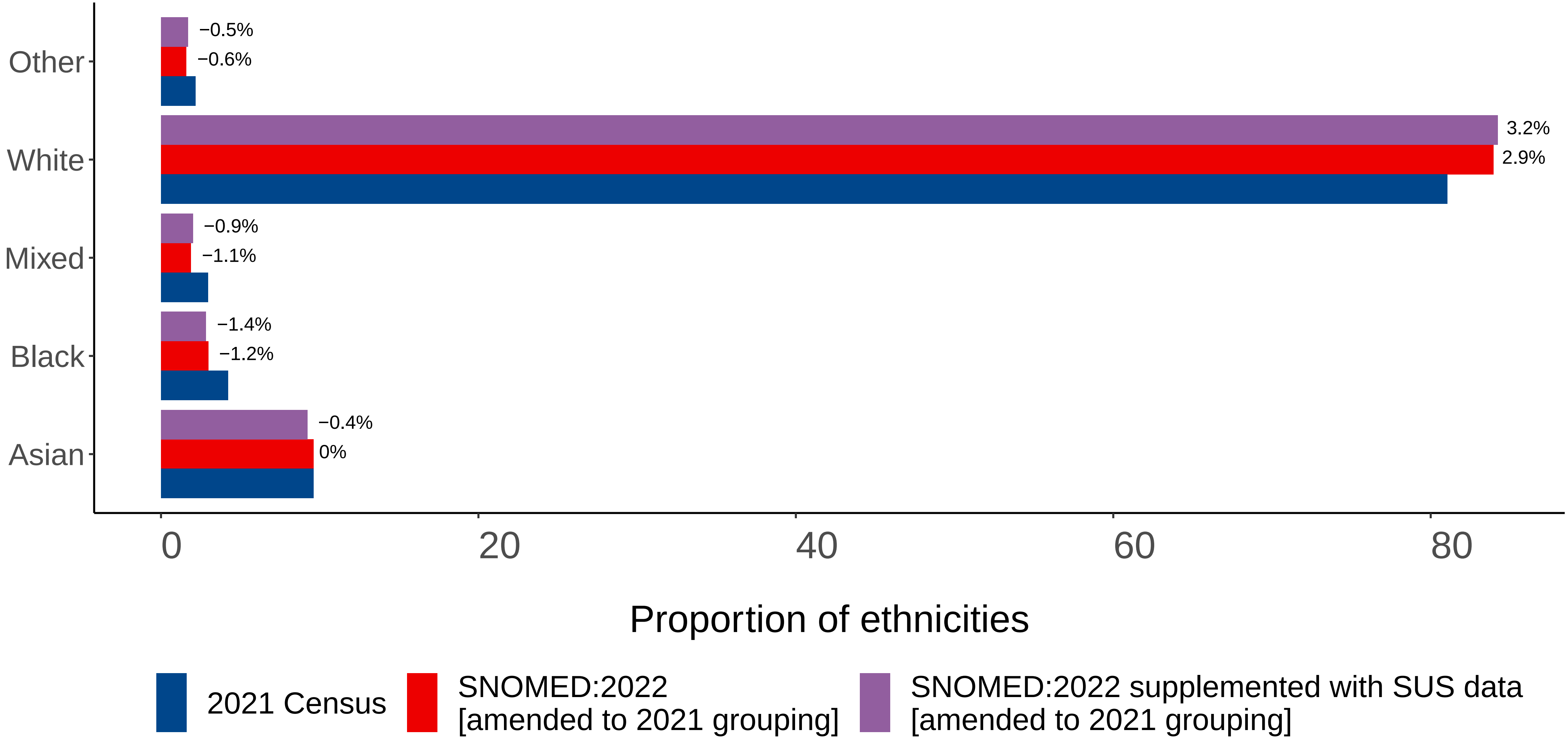

Figure 2: Barplot showing the proportion of 2021 Census and TPP populations (amended to 2021 grouping) per ethnicity grouped into 5 groups per NUTS−1 region (excluding those without a recorded ethnicity). Annotated with percentage point difference between 2021 Census and TPP populations.

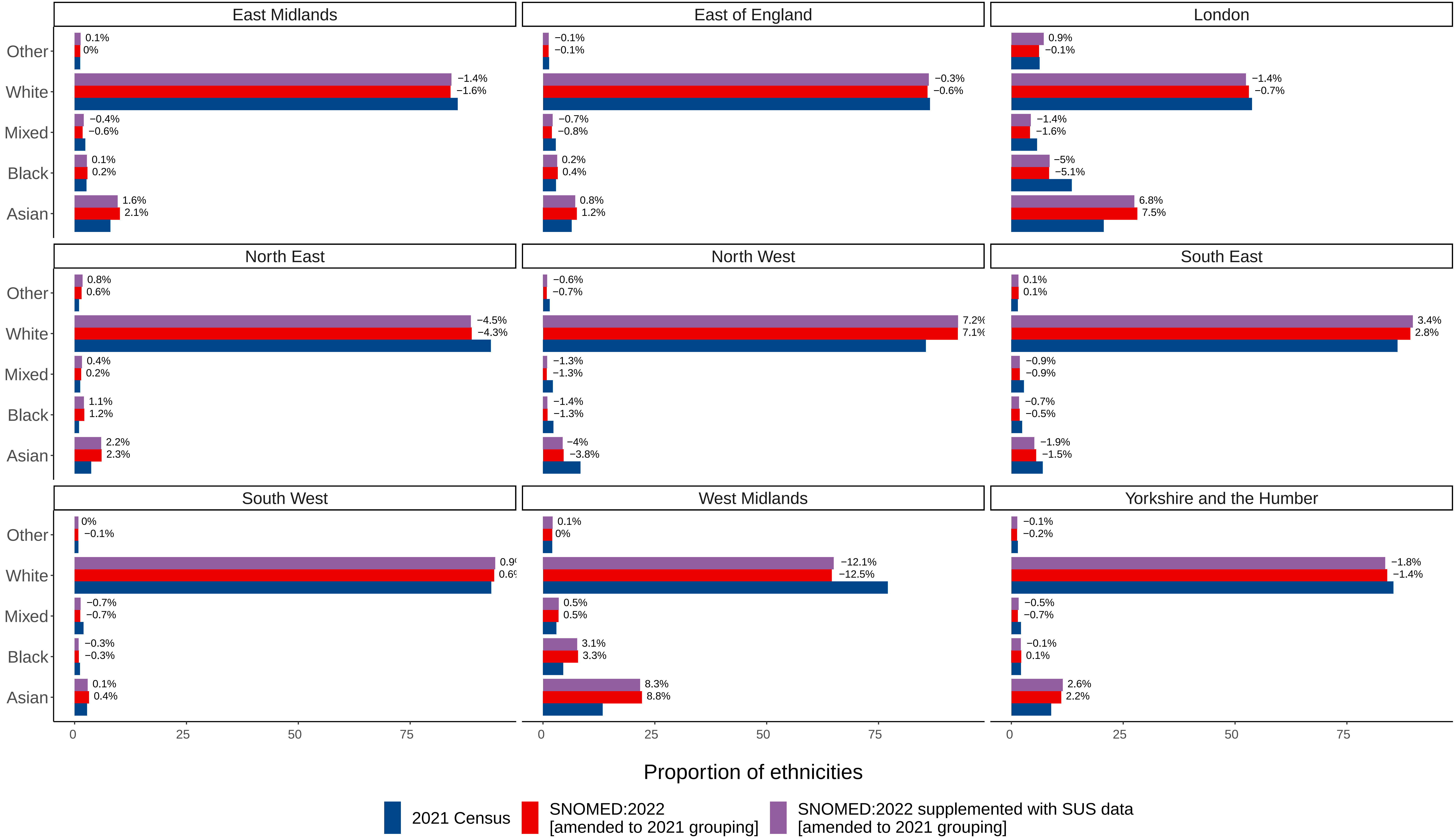

Figure 3: Recording of ethnicity over time for latest and first recorded ethnicity. Unknown dates of recording may be stored as '1900−01−01'

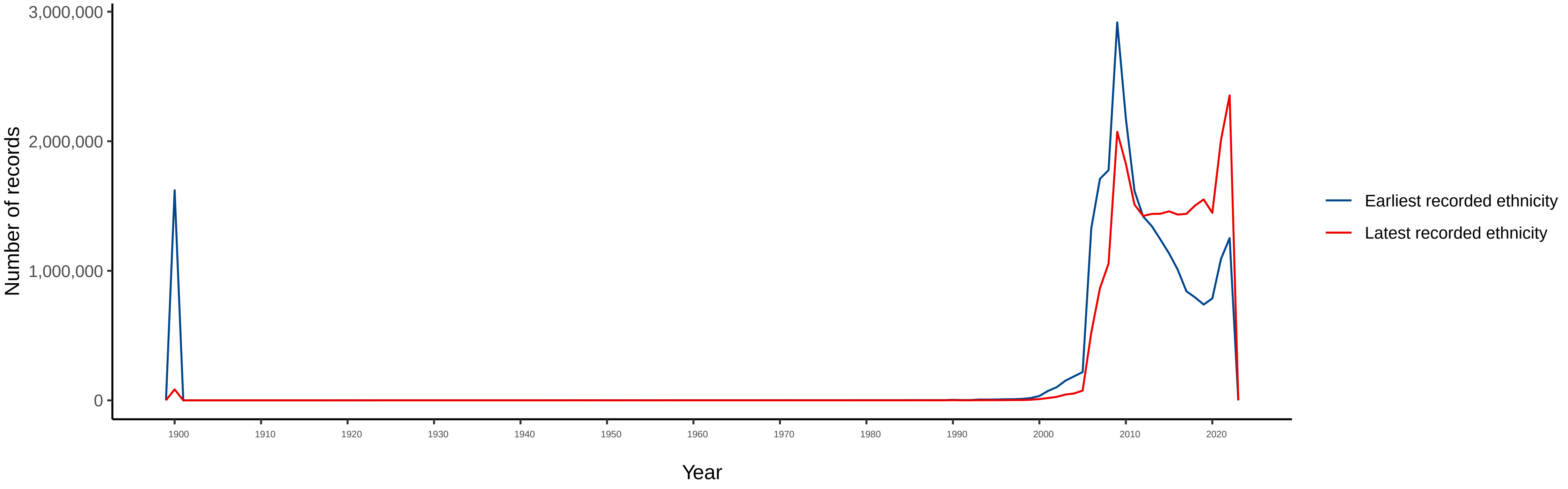

Table 1: Count of patients with a recorded ethnicity in OpenSAFELY-TPP (proportion of registered TPP population) by clinical and demographic subgroups. All counts are rounded to the nearest 5.

	SNOMED 2022	SNOMED 2022 with SUS data	Population	Percentage point increase with SUS data
all				
with records	19,618,135 (78.2)	23,228,760 (92.5)	25,102,210	(14.3)
age band				
0-19	3,506,695 (63.6)	5,019,200 (91.1)	5,509,975	(27.5)
20-29	2,300,970 (73.6)	2,767,405 (88.5)	3,128,250	(14.9)
30-39	2,985,455 (83.0)	3,327,535 (92.5)	3,597,050	(9.5)
40-49	2,730,080 (84.5)	2,989,030 (92.6)	3,228,995	(8.1)
50-59	2,887,720 (83.9)	3,199,355 (93.0)	3,440,755	(9.1)
60-69	2,326,765 (84.9)	2,589,095 (94.5)	2,741,010	(9.6)
70-79	1,875,495 (85.2)	2,118,495 (96.2)	2,201,400	(11.0)
80+	1,004,955 (80.1)	1,218,635 (97.1)	1,254,775	(17.0)
sex				
Female	10,005,930 (79.8)	11,782,895 (94.0)	12,532,940	(14.2)
Male	9,612,205 (76.5)	11,445,860 (91.1)	12,569,270	(14.6)
region				
East	4,488,910 (77.3)	5,355,465 (92.2)	5,808,670	(14.9)
East Midlands	3,413,070 (78.6)	4,020,145 (92.6)	4,343,010	(14.0)
London	1,460,750 (81.4)	1,629,015 (90.7)	1,795,300	(9.3)
North East	914,095 (78.0)	1,103,540 (94.2)	1,171,695	(16.2)
North West	1,693,835 (78.4)	2,029,735 (94.0)	2,159,325	(15.6)
South East	1,265,915 (77.0)	1,502,860 (91.4)	1,644,505	(14.4)
South West	2,697,960 (77.4)	3,219,590 (92.4)	3,483,925	(15.0)
West Midlands	837,230 (82.2)	947,725 (93.0)	1,018,680	(10.8)
Yorkshire and The Humber	2,816,935 (77.4)	3,385,170 (93.0)	3,638,855	(15.6)
IMD				
1 Most deprived	3,885,360 (78.2)	4,633,110 (93.3)	4,968,330	(15.1)
2	3,821,805 (78.5)	4,515,210 (92.8)	4,868,115	(14.3)
3	4,027,315 (78.9)	4,724,895 (92.6)	5,102,160	(13.7)
4	3,871,300 (78.3)	4,559,785 (92.3)	4,942,265	(14.0)
5 Least deprived	3,482,535 (77.7)	4,116,455 (91.8)	4,481,755	(14.1)
Unknown	529,815 (71.6)	679,300 (91.8)	739,585	(20.2)
dementia				
Present	36,645 (82.2)	44,075 (98.9)	44,560	(16.7)
Absent	19,581,490 (78.1)	23,184,680 (92.5)	25,057,655	(14.4)
diabetes				
Present	2,049,495 (85.6)	2,335,080 (97.5)	2,394,370	(11.9)
Absent	17,568,640 (77.4)	20,893,675 (92.0)	22,707,845	(14.6)
hypertension				
Present	1,621,375 (86.0)	1,833,885 (97.2)	1,886,165	(11.2)
Absent	17,996,755 (77.5)	21,394,870 (92.2)	23,216,045	(14.7)
learning disability				
Present	120,385 (84.2)	137,380 (96.1)	143,025	(11.9)
Absent	19,497,750 (78.1)	23,091,375 (92.5)	24,959,185	(14.4)

Table 2: Count of patients with a recorded ethnicity in OpenSAFELY TPP by ethnicity group (proportion of registered TPP population) and clinical and demographic subgroups. All counts are rounded to the nearest 5.

		Asian		Black		Mixed		White		Other	
		**SNOMED 2022**	**SNOMED 2022 with SUS data**	**SNOMED 2022**	**SNOMED 2022 with SUS data**	**SNOMED 2022**	**SNOMED 2022 with SUS data**	**SNOMED 2022**	**SNOMED 2022 with SUS data**	**SNOMED 2022**	**SNOMED 2022 with SUS data**
all	with records	1,708,430 (6.8)	1,955,095 (7.8)	583,770 (2.3)	659,410 (2.6)	367,980 (1.5)	466,220 (1.9)	16,468,610 (65.6)	19,566,635 (77.9)	489,350 (1.9)	581,395 (2.3)
age band	0-19	383,285 (7.0)	536,695 (9.7)	138,355 (2.5)	182,015 (3.3)	137,175 (2.5)	210,360 (3.8)	2,754,320 (50.0)	3,951,305 (71.7)	93,560 (1.7)	138,830 (2.5)
	20-29	259,930 (8.3)	289,485 (9.3)	86,955 (2.8)	95,870 (3.1)	63,215 (2.0)	72,340 (2.3)	1,775,885 (56.8)	2,180,550 (69.7)	114,985 (3.7)	129,165 (4.1)
	30-39	363,460 (10.1)	386,210 (10.7)	102,485 (2.8)	109,505 (3.0)	64,395 (1.8)	70,745 (2.0)	2,350,510 (65.3)	2,644,670 (73.5)	104,605 (2.9)	116,405 (3.2)
	40-49	315,825 (9.8)	331,920 (10.3)	104,610 (3.2)	109,780 (3.4)	46,475 (1.4)	49,975 (1.5)	2,181,060 (67.5)	2,407,950 (74.6)	82,105 (2.5)	89,405 (2.8)
	50-59	182,030 (5.3)	192,220 (5.6)	85,405 (2.5)	90,855 (2.6)	31,895 (0.9)	34,685 (1.0)	2,539,560 (73.8)	2,827,325 (82.2)	48,835 (1.4)	54,280 (1.6)
	60-69	116,945 (4.3)	124,535 (4.5)	40,280 (1.5)	43,280 (1.6)	15,365 (0.6)	16,995 (0.6)	2,126,970 (77.6)	2,373,055 (86.6)	27,200 (1.0)	31,230 (1.1)
	70-79	58,290 (2.6)	62,645 (2.8)	14,745 (0.7)	15,960 (0.7)	6,290 (0.3)	7,285 (0.3)	1,783,385 (81.0)	2,017,300 (91.6)	12,785 (0.6)	15,305 (0.7)
	80+	28,670 (2.3)	31,390 (2.5)	10,930 (0.9)	12,145 (1.0)	3,165 (0.3)	3,840 (0.3)	956,915 (76.3)	1,164,480 (92.8)	5,270 (0.4)	6,780 (0.5)
sex	Female	827,980 (6.6)	949,050 (7.6)	291,010 (2.3)	328,460 (2.6)	186,680 (1.5)	234,280 (1.9)	8,461,075 (67.5)	9,987,690 (79.7)	239,185 (1.9)	283,420 (2.3)
	Male	880,450 (7.0)	1,006,045 (8.0)	292,760 (2.3)	330,955 (2.6)	181,300 (1.4)	231,940 (1.8)	8,007,535 (63.7)	9,578,950 (76.2)	250,160 (2.0)	297,980 (2.4)
region	East	300,605 (5.2)	342,735 (5.9)	147,865 (2.5)	168,770 (2.9)	89,825 (1.5)	116,180 (2.0)	3,856,555 (66.4)	4,617,355 (79.5)	94,055 (1.6)	110,425 (1.9)
	East Midlands	319,445 (7.4)	360,305 (8.3)	97,320 (2.2)	110,695 (2.5)	60,985 (1.4)	81,860 (1.9)	2,867,445 (66.0)	3,386,935 (78.0)	67,880 (1.6)	80,350 (1.9)
	London	368,555 (20.5)	403,495 (22.5)	123,080 (6.9)	138,875 (7.7)	60,995 (3.4)	70,810 (3.9)	775,090 (43.2)	853,735 (47.6)	133,035 (7.4)	162,100 (9.0)
	North East	48,500 (4.1)	58,495 (5.0)	19,950 (1.7)	22,990 (2.0)	13,465 (1.1)	18,160 (1.5)	811,235 (69.2)	977,415 (83.4)	20,940 (1.8)	26,485 (2.3)
	North West	68,150 (3.2)	78,725 (3.6)	17,470 (0.8)	19,705 (0.9)	14,420 (0.7)	19,055 (0.9)	1,570,265 (72.7)	1,882,895 (87.2)	23,535 (1.1)	29,350 (1.4)
	South East	59,670 (3.6)	66,180 (4.0)	23,510 (1.4)	25,550 (1.6)	23,715 (1.4)	28,355 (1.7)	1,128,545 (68.6)	1,348,710 (82.0)	30,470 (1.9)	34,070 (2.1)
	South West	71,795 (2.1)	78,600 (2.3)	25,735 (0.7)	28,730 (0.8)	33,950 (1.0)	42,945 (1.2)	2,529,835 (72.6)	3,026,520 (86.9)	36,640 (1.1)	42,795 (1.2)
	West Midlands	174,370 (17.1)	194,405 (19.1)	65,400 (6.4)	72,195 (7.1)	29,220 (2.9)	33,420 (3.3)	540,185 (53.0)	615,885 (60.5)	28,055 (2.8)	31,815 (3.1)
	Yorkshire and The Humber	294,930 (8.1)	369,235 (10.1)	62,195 (1.7)	70,370 (1.9)	40,435 (1.1)	54,080 (1.5)	2,365,245 (65.0)	2,828,245 (77.7)	54,135 (1.5)	63,245 (1.7)
IMD	1 Most deprived	552,360 (11.1)	646,705 (13.0)	232,370 (4.7)	262,400 (5.3)	100,090 (2.0)	127,030 (2.6)	2,882,250 (58.0)	3,452,775 (69.5)	118,290 (2.4)	144,200 (2.9)
	2	460,265 (9.5)	521,460 (10.7)	143,240 (2.9)	161,860 (3.3)	79,195 (1.6)	99,685 (2.0)	3,029,195 (62.2)	3,600,725 (74.0)	109,910 (2.3)	131,485 (2.7)
	3	312,525 (6.1)	349,530 (6.9)	91,495 (1.8)	102,700 (2.0)	68,860 (1.3)	86,075 (1.7)	3,460,745 (67.8)	4,076,195 (79.9)	93,695 (1.8)	110,400 (2.2)
	4	197,745 (4.0)	222,365 (4.5)	59,885 (1.2)	67,155 (1.4)	59,330 (1.2)	74,685 (1.5)	3,468,920 (70.2)	4,096,445 (82.9)	85,420 (1.7)	99,135 (2.0)
	5 Least deprived	142,175 (3.2)	160,720 (3.6)	37,805 (0.8)	42,430 (0.9)	48,195 (1.1)	61,140 (1.4)	3,190,305 (71.2)	3,777,735 (84.3)	64,055 (1.4)	74,435 (1.7)
	Unknown	43,360 (5.9)	54,320 (7.3)	18,970 (2.6)	22,865 (3.1)	12,310 (1.7)	17,610 (2.4)	437,195 (59.1)	562,760 (76.1)	17,980 (2.4)	21,740 (2.9)
dementia	Present	1,325 (3.0)	1,455 (3.3)	625 (1.4)	705 (1.6)	155 (0.3)	180 (0.4)	34,360 (77.1)	41,485 (93.1)	180 (0.4)	250 (0.6)
	Absent	1,707,105 (6.8)	1,953,640 (7.8)	583,140 (2.3)	658,705 (2.6)	367,820 (1.5)	466,040 (1.9)	16,434,250 (65.6)	19,525,150 (77.9)	489,170 (2.0)	581,145 (2.3)
diabetes	Present	275,225 (11.5)	294,210 (12.3)	73,500 (3.1)	78,875 (3.3)	23,905 (1.0)	26,450 (1.1)	1,643,120 (68.6)	1,896,130 (79.2)	33,740 (1.4)	39,415 (1.6)
	Absent	1,433,205 (6.3)	1,660,885 (7.3)	510,265 (2.2)	580,540 (2.6)	344,075 (1.5)	439,770 (1.9)	14,825,490 (65.3)	17,670,505 (77.8)	455,605 (2.0)	541,980 (2.4)
hypertension	Present	77,420 (4.1)	82,950 (4.4)	35,695 (1.9)	38,290 (2.0)	11,145 (0.6)	12,420 (0.7)	1,483,585 (78.7)	1,684,145 (89.3)	13,535 (0.7)	16,085 (0.9)
	Absent	1,631,010 (7.0)	1,872,140 (8.1)	548,075 (2.4)	621,125 (2.7)	356,835 (1.5)	453,800 (2.0)	14,985,025 (64.5)	17,882,495 (77.0)	475,815 (2.0)	565,315 (2.4)
learning disability	Present	8,450 (5.9)	9,750 (6.8)	2,745 (1.9)	3,085 (2.2)	2,000 (1.4)	2,450 (1.7)	106,145 (74.2)	120,710 (84.4)	1,040 (0.7)	1,385 (1.0)
	Absent	1,699,980 (6.8)	1,945,345 (7.8)	581,020 (2.3)	656,325 (2.6)	365,980 (1.5)	463,770 (1.9)	16,362,460 (65.6)	19,445,925 (77.9)	488,310 (2.0)	580,010 (2.3)

Table 3: Count of patients with a recorded ethnicity in OpenSAFELY TPP by ethnicity group (proportion of registered TPP population) and clinical and demographic subgroups. All counts are rounded to the nearest 5.

		Indian		Pakistani		Bangladeshi		Other Asian		Caribbean		African		Other Black		White and Black Caribbean	
		**SNOMED 2022**	**SNOMED 2022 with SUS data**	**SNOMED 2022**	**SNOMED 2022 with SUS data**	**SNOMED 2022**	**SNOMED 2022 with SUS data**	**SNOMED 2022**	**SNOMED 2022 with SUS data**	**SNOMED 2022**	**SNOMED 2022 with SUS data**	**SNOMED 2022**	**SNOMED 2022 with SUS data**	**SNOMED 2022**	**SNOMED 2022 with SUS data**	**SNOMED 2022**	**SNOMED 2022 with SUS data**
all	with records	690,425 (2.8)	765,870 (3.1)	499,570 (2.0)	604,255 (2.4)	120,610 (0.5)	139,920 (0.6)	397,825 (1.6)	445,050 (1.8)	113,575 (0.5)	132,025 (0.5)	369,315 (1.5)	410,730 (1.6)	100,880 (0.4)	116,705 (0.5)	82,660 (0.3)	105,695 (0.4)
age band	0-19	123,800 (2.2)	169,220 (3.1)	143,275 (2.6)	211,650 (3.8)	34,080 (0.6)	46,840 (0.9)	82,130 (1.5)	108,970 (2.0)	15,525 (0.3)	22,640 (0.4)	95,135 (1.7)	124,240 (2.3)	27,690 (0.5)	35,135 (0.6)	30,755 (0.6)	47,570 (0.9)
	20-29	101,930 (3.3)	108,965 (3.5)	74,360 (2.4)	88,700 (2.8)	19,345 (0.6)	21,455 (0.7)	64,300 (2.1)	70,410 (2.3)	12,965 (0.4)	15,680 (0.5)	58,895 (1.9)	62,305 (2.0)	15,095 (0.5)	17,895 (0.6)	15,235 (0.5)	17,995 (0.6)
	30-39	153,680 (4.3)	160,435 (4.5)	99,185 (2.8)	108,270 (3.0)	24,670 (0.7)	26,230 (0.7)	85,920 (2.4)	91,280 (2.5)	16,155 (0.4)	18,255 (0.5)	70,220 (2.0)	73,295 (2.0)	16,110 (0.4)	17,970 (0.5)	14,355 (0.4)	16,150 (0.4)
	40-49	128,255 (4.0)	133,280 (4.1)	86,895 (2.7)	92,995 (2.9)	23,290 (0.7)	24,635 (0.8)	77,390 (2.4)	80,990 (2.5)	15,920 (0.5)	17,315 (0.5)	72,850 (2.3)	75,325 (2.3)	15,840 (0.5)	17,155 (0.5)	7,905 (0.2)	8,595 (0.3)
	50-59	76,600 (2.2)	80,550 (2.3)	47,805 (1.4)	50,855 (1.5)	10,960 (0.3)	11,735 (0.3)	46,665 (1.4)	49,070 (1.4)	23,330 (0.7)	25,530 (0.7)	46,385 (1.3)	48,305 (1.4)	15,690 (0.5)	17,025 (0.5)	7,800 (0.2)	8,320 (0.2)
	60-69	58,970 (2.2)	62,460 (2.3)	28,355 (1.0)	30,445 (1.1)	5,135 (0.2)	5,555 (0.2)	24,485 (0.9)	26,075 (1.0)	15,745 (0.6)	17,165 (0.6)	17,505 (0.6)	18,440 (0.7)	7,030 (0.3)	7,685 (0.3)	3,900 (0.1)	4,140 (0.2)
	70-79	31,840 (1.4)	34,180 (1.6)	12,275 (0.6)	13,220 (0.6)	1,875 (0.1)	2,065 (0.1)	12,300 (0.6)	13,180 (0.6)	6,565 (0.3)	7,195 (0.3)	5,920 (0.3)	6,255 (0.3)	2,260 (0.1)	2,515 (0.1)	1,475 (0.1)	1,580 (0.1)
	80+	15,355 (1.2)	16,785 (1.3)	7,425 (0.6)	8,120 (0.6)	1,255 (0.1)	1,410 (0.1)	4,635 (0.4)	5,075 (0.4)	7,370 (0.6)	8,245 (0.7)	2,400 (0.2)	2,570 (0.2)	1,160 (0.1)	1,335 (0.1)	1,230 (0.1)	1,335 (0.1)
sex	Female	326,795 (2.6)	363,830 (2.9)	242,560 (1.9)	293,875 (2.3)	57,715 (0.5)	67,620 (0.5)	200,910 (1.6)	223,735 (1.8)	59,055 (0.5)	68,255 (0.5)	181,750 (1.5)	202,745 (1.6)	50,205 (0.4)	57,485 (0.5)	42,970 (0.3)	54,485 (0.4)
	Male	363,630 (2.9)	402,040 (3.2)	257,005 (2.0)	310,380 (2.5)	62,895 (0.5)	72,300 (0.6)	196,915 (1.6)	221,315 (1.8)	54,520 (0.4)	63,770 (0.5)	187,565 (1.5)	207,990 (1.7)	50,675 (0.4)	59,220 (0.5)	39,690 (0.3)	51,205 (0.4)
region	East	108,030 (1.9)	120,340 (2.1)	80,525 (1.4)	94,650 (1.6)	36,895 (0.6)	43,115 (0.7)	75,155 (1.3)	84,630 (1.5)	23,740 (0.4)	28,380 (0.5)	96,865 (1.7)	109,045 (1.9)	27,260 (0.5)	31,360 (0.5)	18,975 (0.3)	25,250 (0.4)
	East Midlands	200,645 (4.6)	224,035 (5.2)	50,290 (1.2)	58,690 (1.4)	13,355 (0.3)	15,670 (0.4)	55,155 (1.3)	61,890 (1.4)	18,280 (0.4)	21,695 (0.5)	63,970 (1.5)	71,285 (1.6)	15,070 (0.3)	17,695 (0.4)	18,405 (0.4)	24,435 (0.6)
	London	173,335 (9.7)	188,780 (10.5)	51,010 (2.8)	56,005 (3.1)	17,725 (1.0)	19,475 (1.1)	126,490 (7.0)	139,255 (7.8)	27,865 (1.6)	31,590 (1.8)	74,260 (4.1)	82,600 (4.6)	20,960 (1.2)	24,725 (1.4)	9,025 (0.5)	10,515 (0.6)
	North East	8,640 (0.7)	9,720 (0.8)	25,385 (2.2)	31,835 (2.7)	2,610 (0.2)	3,260 (0.3)	11,860 (1.0)	13,675 (1.2)	2,675 (0.2)	3,270 (0.3)	14,750 (1.3)	16,560 (1.4)	2,525 (0.2)	3,160 (0.3)	3,445 (0.3)	4,600 (0.4)
	North West	23,000 (1.1)	25,160 (1.2)	21,165 (1.0)	25,745 (1.2)	6,705 (0.3)	7,975 (0.4)	17,280 (0.8)	19,865 (0.9)	1,135 (0.1)	1,260 (0.1)	13,410 (0.6)	14,960 (0.7)	2,925 (0.1)	3,495 (0.2)	1,530 (0.1)	2,025 (0.1)
	South East	21,610 (1.3)	23,470 (1.4)	9,420 (0.6)	10,780 (0.7)	7,455 (0.5)	8,425 (0.5)	21,190 (1.3)	23,495 (1.4)	2,825 (0.2)	3,085 (0.2)	16,005 (1.0)	17,215 (1.0)	4,675 (0.3)	5,255 (0.3)	3,385 (0.2)	4,015 (0.2)
	South West	33,820 (1.0)	36,535 (1.0)	4,945 (0.1)	5,535 (0.2)	5,095 (0.1)	5,935 (0.2)	27,940 (0.8)	30,595 (0.9)	4,815 (0.1)	5,555 (0.2)	15,330 (0.4)	16,685 (0.5)	5,590 (0.2)	6,485 (0.2)	7,080 (0.2)	8,780 (0.3)
	West Midlands	65,260 (6.4)	73,425 (7.2)	70,250 (6.9)	77,920 (7.6)	15,380 (1.5)	17,375 (1.7)	23,485 (2.3)	25,695 (2.5)	22,860 (2.2)	26,040 (2.6)	31,365 (3.1)	33,885 (3.3)	11,175 (1.1)	12,265 (1.2)	11,545 (1.1)	13,395 (1.3)
	Yorkshire and The Humber	54,790 (1.5)	62,915 (1.7)	185,960 (5.1)	242,270 (6.7)	15,340 (0.4)	18,625 (0.5)	38,840 (1.1)	45,415 (1.2)	9,200 (0.3)	10,910 (0.3)	42,505 (1.2)	47,490 (1.3)	10,490 (0.3)	11,980 (0.3)	8,965 (0.2)	12,230 (0.3)
IMD	1 Most deprived	137,995 (2.8)	152,740 (3.1)	257,980 (5.2)	314,575 (6.3)	54,780 (1.1)	63,695 (1.3)	101,605 (2.0)	115,695 (2.3)	44,070 (0.9)	51,275 (1.0)	152,360 (3.1)	169,330 (3.4)	35,940 (0.7)	41,805 (0.8)	27,090 (0.5)	34,760 (0.7)
	2	210,115 (4.3)	231,525 (4.8)	115,185 (2.4)	138,270 (2.8)	31,380 (0.6)	36,080 (0.7)	103,580 (2.1)	115,590 (2.4)	27,995 (0.6)	32,675 (0.7)	90,415 (1.9)	100,355 (2.1)	24,830 (0.5)	28,865 (0.6)	18,200 (0.4)	23,165 (0.5)
	3	149,150 (2.9)	164,265 (3.2)	62,685 (1.2)	73,410 (1.4)	18,110 (0.4)	20,705 (0.4)	82,580 (1.6)	91,160 (1.8)	19,805 (0.4)	22,805 (0.4)	54,800 (1.1)	60,490 (1.2)	16,885 (0.3)	19,395 (0.4)	14,925 (0.3)	18,820 (0.4)
	4	96,690 (2.0)	107,600 (2.2)	33,760 (0.7)	40,015 (0.8)	8,920 (0.2)	10,370 (0.2)	58,375 (1.2)	64,390 (1.3)	11,930 (0.2)	13,705 (0.3)	35,890 (0.7)	39,760 (0.8)	12,065 (0.2)	13,700 (0.3)	11,180 (0.2)	14,180 (0.3)
	5 Least deprived	76,390 (1.7)	86,140 (1.9)	20,115 (0.4)	23,595 (0.5)	5,095 (0.1)	6,025 (0.1)	40,570 (0.9)	44,930 (1.0)	7,100 (0.2)	8,215 (0.2)	22,800 (0.5)	25,220 (0.6)	7,905 (0.2)	9,000 (0.2)	8,430 (0.2)	10,720 (0.2)
	Unknown	20,090 (2.7)	23,605 (3.2)	9,840 (1.3)	14,390 (1.9)	2,320 (0.3)	3,040 (0.4)	11,110 (1.5)	13,285 (1.8)	2,670 (0.4)	3,350 (0.5)	13,045 (1.8)	15,575 (2.1)	3,255 (0.4)	3,945 (0.5)	2,835 (0.4)	4,045 (0.5)
dementia	Present	640 (1.4)	695 (1.6)	410 (0.9)	445 (1.0)	100 (0.2)	115 (0.3)	170 (0.4)	205 (0.5)	405 (0.9)	460 (1.0)	150 (0.3)	160 (0.4)	75 (0.2)	80 (0.2)	75 (0.2)	80 (0.2)
	Absent	689,785 (2.8)	765,175 (3.1)	499,160 (2.0)	603,810 (2.4)	120,510 (0.5)	139,810 (0.6)	397,650 (1.6)	444,845 (1.8)	113,170 (0.5)	131,565 (0.5)	369,165 (1.5)	410,570 (1.6)	100,805 (0.4)	116,625 (0.5)	82,585 (0.3)	105,610 (0.4)
diabetes	Present	118,950 (5.0)	126,110 (5.3)	80,610 (3.4)	87,210 (3.6)	22,455 (0.9)	24,220 (1.0)	53,210 (2.2)	56,680 (2.4)	23,810 (1.0)	26,090 (1.1)	38,955 (1.6)	40,880 (1.7)	10,735 (0.4)	11,920 (0.5)	6,155 (0.3)	6,685 (0.3)
	Absent	571,480 (2.5)	639,760 (2.8)	418,955 (1.8)	517,045 (2.3)	98,155 (0.4)	115,705 (0.5)	344,615 (1.5)	388,370 (1.7)	89,760 (0.4)	105,935 (0.5)	330,360 (1.5)	369,850 (1.6)	90,145 (0.4)	104,785 (0.5)	76,505 (0.3)	99,010 (0.4)
hypertension	Present	33,900 (1.8)	36,065 (1.9)	21,680 (1.1)	23,480 (1.2)	5,350 (0.3)	5,815 (0.3)	16,485 (0.9)	17,580 (0.9)	11,365 (0.6)	12,500 (0.7)	18,705 (1.0)	19,620 (1.0)	5,625 (0.3)	6,180 (0.3)	3,055 (0.2)	3,280 (0.2)
	Absent	656,525 (2.8)	729,805 (3.1)	477,885 (2.1)	580,780 (2.5)	115,260 (0.5)	134,105 (0.6)	381,340 (1.6)	427,470 (1.8)	102,210 (0.4)	119,525 (0.5)	350,610 (1.5)	391,110 (1.7)	95,255 (0.4)	110,525 (0.5)	79,605 (0.3)	102,415 (0.4)
learning disability	Present	2,210 (1.5)	2,455 (1.7)	4,145 (2.9)	4,895 (3.4)	725 (0.5)	835 (0.6)	1,370 (1.0)	1,570 (1.1)	830 (0.6)	910 (0.6)	1,235 (0.9)	1,420 (1.0)	685 (0.5)	755 (0.5)	625 (0.4)	735 (0.5)
	Absent	688,215 (2.8)	763,415 (3.1)	495,425 (2.0)	599,360 (2.4)	119,885 (0.5)	139,085 (0.6)	396,455 (1.6)	443,480 (1.8)	112,740 (0.5)	131,115 (0.5)	368,080 (1.5)	409,315 (1.6)	100,195 (0.4)	115,950 (0.5)	82,035 (0.3)	104,960 (0.4)

		White and Black African		White and Asian		Other Mixed		White British		White Irish		Other White		Chinese		Any other ethnic group	
		**SNOMED 2022**	**SNOMED 2022 with SUS data**	**SNOMED 2022**	**SNOMED 2022 with SUS data**	**SNOMED 2022**	**SNOMED 2022 with SUS data**	**SNOMED 2022**	**SNOMED 2022 with SUS data**	**SNOMED 2022**	**SNOMED 2022 with SUS data**	**SNOMED 2022**	**SNOMED 2022 with SUS data**	**SNOMED 2022**	**SNOMED 2022 with SUS data**	**SNOMED 2022**	**SNOMED 2022 with SUS data**
all	with records	68,980 (0.3)	80,555 (0.3)	78,945 (0.3)	99,505 (0.4)	137,400 (0.5)	180,605 (0.7)	14,073,280 (56.1)	16,948,545 (67.5)	109,280 (0.4)	121,220 (0.5)	2,286,050 (9.1)	2,496,700 (9.9)	176,970 (0.7)	186,855 (0.7)	312,375 (1.2)	394,495 (1.6)
age band	0-19	24,225 (0.4)	33,590 (0.6)	31,610 (0.6)	48,215 (0.9)	50,585 (0.9)	81,100 (1.5)	2,364,790 (42.9)	3,441,095 (62.5)	8,280 (0.2)	11,695 (0.2)	381,250 (6.9)	498,520 (9.0)	24,620 (0.4)	29,300 (0.5)	68,940 (1.3)	109,505 (2.0)
	20-29	10,740 (0.3)	11,550 (0.4)	14,230 (0.5)	15,730 (0.5)	23,005 (0.7)	27,060 (0.9)	1,417,355 (45.3)	1,797,380 (57.5)	11,260 (0.4)	12,295 (0.4)	347,270 (11.1)	370,735 (11.9)	61,625 (2.0)	62,780 (2.0)	53,355 (1.7)	66,405 (2.1)
	30-39	11,510 (0.3)	11,990 (0.3)	12,810 (0.4)	13,770 (0.4)	25,720 (0.7)	28,840 (0.8)	1,772,795 (49.3)	2,041,980 (56.8)	16,765 (0.5)	17,705 (0.5)	560,950 (15.6)	584,915 (16.3)	33,710 (0.9)	34,775 (1.0)	70,895 (2.0)	81,630 (2.3)
	40-49	11,125 (0.3)	11,510 (0.4)	9,290 (0.3)	9,830 (0.3)	18,155 (0.6)	20,045 (0.6)	1,749,745 (54.2)	1,960,865 (60.7)	15,540 (0.5)	16,340 (0.5)	415,770 (12.9)	430,825 (13.3)	24,945 (0.8)	25,800 (0.8)	57,160 (1.8)	63,590 (2.0)
	50-59	6,840 (0.2)	7,130 (0.2)	6,140 (0.2)	6,560 (0.2)	11,115 (0.3)	12,690 (0.4)	2,266,255 (65.9)	2,540,685 (73.8)	17,620 (0.5)	18,925 (0.6)	255,685 (7.4)	267,650 (7.8)	16,005 (0.5)	16,745 (0.5)	32,830 (1.0)	37,525 (1.1)
	60-69	2,980 (0.1)	3,140 (0.1)	2,930 (0.1)	3,205 (0.1)	5,555 (0.2)	6,515 (0.2)	1,952,110 (71.2)	2,188,565 (79.8)	15,285 (0.6)	16,515 (0.6)	159,575 (5.8)	167,970 (6.1)	9,935 (0.4)	10,630 (0.4)	17,265 (0.6)	20,605 (0.8)
	70-79	1,160 (0.1)	1,220 (0.1)	1,375 (0.1)	1,550 (0.1)	2,280 (0.1)	2,935 (0.1)	1,663,155 (75.5)	1,889,605 (85.8)	15,065 (0.7)	16,635 (0.8)	105,165 (4.8)	111,065 (5.0)	4,385 (0.2)	4,850 (0.2)	8,400 (0.4)	10,440 (0.5)
	80+	400 (0.0)	425 (0.0)	560 (0.0)	650 (0.1)	975 (0.1)	1,415 (0.1)	887,065 (70.7)	1,088,360 (86.7)	9,465 (0.8)	11,105 (0.9)	60,385 (4.8)	65,015 (5.2)	1,740 (0.1)	1,975 (0.2)	3,530 (0.3)	4,795 (0.4)
sex	Female	34,340 (0.3)	40,070 (0.3)	40,000 (0.3)	50,140 (0.4)	69,370 (0.6)	89,685 (0.7)	7,252,300 (57.9)	8,666,365 (69.1)	54,965 (0.4)	61,080 (0.5)	1,153,810 (9.2)	1,260,105 (10.1)	92,015 (0.7)	97,325 (0.8)	147,170 (1.2)	186,080 (1.5)
	Male	34,640 (0.3)	40,485 (0.3)	38,945 (0.3)	49,365 (0.4)	68,025 (0.5)	90,920 (0.7)	6,820,980 (54.3)	8,282,180 (65.9)	54,315 (0.4)	60,140 (0.5)	1,132,240 (9.0)	1,236,590 (9.8)	84,955 (0.7)	89,530 (0.7)	165,205 (1.3)	208,420 (1.7)
region	East	20,295 (0.3)	24,090 (0.4)	17,785 (0.3)	23,130 (0.4)	32,775 (0.6)	43,720 (0.8)	3,207,190 (55.2)	3,901,430 (67.2)	28,660 (0.5)	32,650 (0.6)	620,700 (10.7)	683,255 (11.8)	38,980 (0.7)	41,610 (0.7)	55,075 (0.9)	68,805 (1.2)
	East Midlands	10,760 (0.2)	12,700 (0.3)	13,030 (0.3)	16,530 (0.4)	18,795 (0.4)	28,235 (0.7)	2,491,890 (57.4)	2,976,340 (68.5)	14,410 (0.3)	16,095 (0.4)	361,140 (8.3)	394,435 (9.1)	24,830 (0.6)	26,205 (0.6)	43,050 (1.0)	54,200 (1.2)
	London	8,735 (0.5)	9,735 (0.5)	11,270 (0.6)	13,110 (0.7)	31,965 (1.8)	37,475 (2.1)	348,760 (19.4)	392,040 (21.8)	22,180 (1.2)	23,850 (1.3)	404,150 (22.5)	437,875 (24.4)	42,690 (2.4)	44,290 (2.5)	90,345 (5.0)	117,735 (6.6)
	North East	2,750 (0.2)	3,305 (0.3)	3,110 (0.3)	4,175 (0.4)	4,160 (0.4)	6,095 (0.5)	742,000 (63.3)	902,025 (77.0)	2,655 (0.2)	3,020 (0.3)	66,585 (5.7)	72,405 (6.2)	6,545 (0.6)	7,065 (0.6)	14,395 (1.2)	19,430 (1.7)
	North West	2,980 (0.1)	3,675 (0.2)	3,995 (0.2)	5,380 (0.2)	5,920 (0.3)	7,985 (0.4)	1,462,975 (67.8)	1,769,575 (82.0)	5,300 (0.2)	5,835 (0.3)	101,985 (4.7)	107,465 (5.0)	9,635 (0.4)	10,390 (0.5)	13,900 (0.6)	18,975 (0.9)
	South East	4,635 (0.3)	5,365 (0.3)	5,805 (0.4)	6,805 (0.4)	9,890 (0.6)	12,170 (0.7)	963,370 (58.6)	1,169,575 (71.1)	7,740 (0.5)	8,565 (0.5)	157,435 (9.6)	170,525 (10.4)	10,125 (0.6)	10,660 (0.6)	20,345 (1.2)	23,400 (1.4)
	South West	5,790 (0.2)	6,845 (0.2)	8,515 (0.2)	10,420 (0.3)	12,570 (0.4)	16,925 (0.5)	2,256,880 (64.8)	2,729,835 (78.4)	11,385 (0.3)	12,675 (0.4)	261,570 (7.5)	284,000 (8.2)	15,010 (0.4)	15,935 (0.5)	21,635 (0.6)	26,855 (0.8)
	West Midlands	3,830 (0.4)	4,160 (0.4)	5,390 (0.5)	6,060 (0.6)	8,460 (0.8)	9,820 (1.0)	461,205 (45.3)	530,980 (52.1)	6,655 (0.7)	7,150 (0.7)	72,325 (7.1)	77,690 (7.6)	10,710 (1.1)	11,205 (1.1)	17,345 (1.7)	20,605 (2.0)
	Yorkshire and The Humber	8,900 (0.2)	10,325 (0.3)	9,895 (0.3)	13,665 (0.4)	12,675 (0.3)	17,850 (0.5)	2,118,540 (58.2)	2,552,035 (70.1)	10,160 (0.3)	11,235 (0.3)	236,545 (6.5)	264,945 (7.3)	18,300 (0.5)	19,335 (0.5)	35,835 (1.0)	43,900 (1.2)
IMD	1 Most deprived	22,300 (0.4)	25,555 (0.5)	17,080 (0.3)	21,885 (0.4)	33,620 (0.7)	44,835 (0.9)	2,329,005 (46.9)	2,848,830 (57.3)	17,825 (0.4)	19,750 (0.4)	535,420 (10.8)	584,140 (11.8)	24,690 (0.5)	26,285 (0.5)	93,600 (1.9)	117,880 (2.4)
	2	15,330 (0.3)	17,890 (0.4)	15,285 (0.3)	19,035 (0.4)	30,380 (0.6)	39,585 (0.8)	2,494,495 (51.2)	3,015,755 (61.9)	21,645 (0.4)	23,995 (0.5)	513,060 (10.5)	560,910 (11.5)	36,285 (0.7)	38,145 (0.8)	73,625 (1.5)	93,370 (1.9)
	3	11,945 (0.2)	13,955 (0.3)	15,340 (0.3)	18,885 (0.4)	26,655 (0.5)	34,455 (0.7)	2,960,575 (58.0)	3,531,960 (69.2)	24,175 (0.5)	26,685 (0.5)	475,990 (9.3)	517,505 (10.1)	36,180 (0.7)	37,995 (0.7)	57,515 (1.1)	72,380 (1.4)
	4	9,725 (0.2)	11,415 (0.2)	14,795 (0.3)	18,500 (0.4)	23,630 (0.5)	30,635 (0.6)	3,039,835 (61.5)	3,629,195 (73.4)	23,555 (0.5)	25,985 (0.5)	405,530 (8.2)	441,190 (8.9)	37,465 (0.8)	39,465 (0.8)	47,955 (1.0)	59,660 (1.2)
	5 Least deprived	7,080 (0.2)	8,425 (0.2)	13,910 (0.3)	17,585 (0.4)	18,775 (0.4)	24,440 (0.5)	2,868,285 (64.0)	3,427,485 (76.5)	19,650 (0.4)	21,920 (0.5)	302,375 (6.7)	328,395 (7.3)	33,070 (0.7)	35,100 (0.8)	30,985 (0.7)	39,325 (0.9)
	Unknown	2,605 (0.4)	3,310 (0.4)	2,530 (0.3)	3,610 (0.5)	4,340 (0.6)	6,655 (0.9)	381,085 (51.5)	495,315 (67.0)	2,430 (0.3)	2,890 (0.4)	53,675 (7.3)	64,560 (8.7)	9,285 (1.3)	9,865 (1.3)	8,695 (1.2)	11,885 (1.6)
dementia	Present	20 (0.0)	20 (0.0)	25 (0.1)	25 (0.1)	35 (0.1)	50 (0.1)	31,845 (71.5)	38,750 (87.0)	405 (0.9)	470 (1.1)	2,110 (4.7)	2,265 (5.1)	45 (0.1)	65 (0.1)	130 (0.3)	180 (0.4)
	Absent	68,960 (0.3)	80,535 (0.3)	78,920 (0.3)	99,480 (0.4)	137,360 (0.5)	180,555 (0.7)	14,041,435 (56.0)	16,909,795 (67.5)	108,875 (0.4)	120,750 (0.5)	2,283,940 (9.1)	2,494,435 (10.0)	176,925 (0.7)	186,795 (0.7)	312,245 (1.2)	394,315 (1.6)
diabetes	Present	5,020 (0.2)	5,310 (0.2)	4,750 (0.2)	5,245 (0.2)	7,985 (0.3)	9,225 (0.4)	1,488,060 (62.1)	1,731,290 (72.3)	12,845 (0.5)	14,215 (0.6)	142,215 (5.9)	150,625 (6.3)	8,935 (0.4)	9,765 (0.4)	24,810 (1.0)	29,635 (1.2)
	Absent	63,960 (0.3)	75,245 (0.3)	74,195 (0.3)	94,260 (0.4)	129,415 (0.6)	171,380 (0.8)	12,585,220 (55.4)	15,217,255 (67.0)	96,435 (0.4)	107,005 (0.5)	2,143,835 (9.4)	2,346,070 (10.3)	168,040 (0.7)	177,095 (0.8)	287,570 (1.3)	364,860 (1.6)
hypertension	Present	2,725 (0.1)	2,865 (0.2)	1,865 (0.1)	2,045 (0.1)	3,495 (0.2)	4,225 (0.2)	1,367,220 (72.5)	1,561,030 (82.8)	10,010 (0.5)	11,095 (0.6)	106,360 (5.6)	112,005 (5.9)	4,240 (0.2)	4,615 (0.2)	9,295 (0.5)	11,470 (0.6)
	Absent	66,250 (0.3)	77,685 (0.3)	77,075 (0.3)	97,460 (0.4)	133,905 (0.6)	176,380 (0.8)	12,706,065 (54.7)	15,387,515 (66.3)	99,270 (0.4)	110,125 (0.5)	2,179,690 (9.4)	2,384,690 (10.3)	172,730 (0.7)	182,245 (0.8)	303,080 (1.3)	383,030 (1.6)
learning disability	Present	295 (0.2)	350 (0.2)	355 (0.2)	445 (0.3)	725 (0.5)	920 (0.6)	99,135 (69.3)	113,130 (79.1)	405 (0.3)	440 (0.3)	6,610 (4.6)	7,145 (5.0)	215 (0.2)	240 (0.2)	825 (0.6)	1,140 (0.8)
	Absent	68,685 (0.3)	80,205 (0.3)	78,590 (0.3)	99,060 (0.4)	136,675 (0.5)	179,685 (0.7)	13,974,145 (56.0)	16,835,410 (67.5)	108,875 (0.4)	120,780 (0.5)	2,279,440 (9.1)	2,489,550 (10.0)	176,760 (0.7)	186,615 (0.7)	311,550 (1.2)	393,355 (1.6)

Table 4: Count of patients' most frequently recorded ethnicity (proportion of latest ethnicity).

Latest Recorded Ethnicity	Most Frequent Ethnicity				
	Asian	Black	Mixed	White	Other
Asian	1,695,745 (99.3)	1,165 (0.1)	1,995 (0.1)	4,690 (0.3)	4,835 (0.3)
Black	4,380 (0.8)	569,290 (97.5)	3,825 (0.7)	5,450 (0.9)	825 (0.1)
Mixed	15,155 (4.1)	26,300 (7.1)	309,220 (84.0)	15,080 (4.1)	2,225 (0.6)
White	23,975 (0.1)	16,845 (0.1)	32,920 (0.2)	16,390,425 (99.5)	4,445 (0.0)
Other	23,970 (4.9)	6,825 (1.4)	9,520 (1.9)	49,600 (10.1)	399,440 (81.6)

Table 5: Count of patients with a recorded ethnicity in Secondary Care by ethnicity group (proportion of Primary Care population). All counts are rounded to the nearest 5.

Primary Care ethnicity	Secondary Care ethnicity					
	**Asian**	**Black**	**Mixed**	**White**	**Other**	**Unknown**
Asian (1,708,430)	822,555 (48.1)	6,935 (0.4)	23,025 (1.3)	37,000 (2.2)	85,035 (5)	733,885 (43)
Black (583,770)	9,080 (1.6)	249,215 (42.7)	23,625 (4)	22,660 (3.9)	27,875 (4.8)	251,315 (43.1)
Mixed (367,980)	19,015 (5.2)	34,550 (9.4)	75,460 (20.5)	62,030 (16.9)	25,640 (7)	151,280 (41.1)
White (16,468,610)	39,165 (0.2)	29,200 (0.2)	105,610 (0.6)	10,871,855 (66)	173,795 (1.1)	5,248,985 (31.9)
Other (489,350)	32,475 (6.6)	6,980 (1.4)	12,575 (2.6)	59,635 (12.2)	91,125 (18.6)	286,555 (58.6)
Unknown (5,484,075)	246,665 (4.5)	75,645 (1.4)	98,240 (1.8)	3,098,030 (56.5)	92,050 (1.7)	1,873,455 (34.2)

Table 6: Count of patients with a recorded ethnicity in Secondary Care by ethnicity group excluding Unknown ethnicites (proportion of Primary Care population). All counts are rounded to the nearest 5.

Primary Care ethnicity	Secondary Care ethnicity				
	**Asian**	**Black**	**Mixed**	**White**	**Other**
Asian (1,708,430)	822,555 (48.1)	6,935 (0.4)	23,025 (1.3)	37,000 (2.2)	85,035 (5)
Black (583,770)	9,080 (1.6)	249,215 (42.7)	23,625 (4)	22,660 (3.9)	27,875 (4.8)
Mixed (367,980)	19,015 (5.2)	34,550 (9.4)	75,460 (20.5)	62,030 (16.9)	25,640 (7)
White (16,468,610)	39,165 (0.2)	29,200 (0.2)	105,610 (0.6)	10,871,855 (66)	173,795 (1.1)
Other (489,350)	32,475 (6.6)	6,980 (1.4)	12,575 (2.6)	59,635 (12.2)	91,125 (18.6)

Table 7: Count of patients with a recorded ethnicity in OpenSAFELY TPP by ethnicity group (proportion of registered TPP population) and 2021 ONS Census counts [amended to 2001 grouping] (proportion of 2021 ONS Census population). All counts are rounded to the nearest 5.

	Asian			Black			Mixed			White			Other		
	**SNOMED 2022**	**SNOMED 2022 with SUS data**	**2021 ONS Census [amended to 2001 grouping]**	**SNOMED 2022**	**SNOMED 2022 with SUS data**	**2021 ONS Census [amended to 2001 grouping]**	**SNOMED 2022**	**SNOMED 2022 with SUS data**	**2021 ONS Census [amended to 2001 grouping]**	**SNOMED 2022**	**SNOMED 2022 with SUS data**	**2021 ONS Census [amended to 2001 grouping]**	**SNOMED 2022**	**SNOMED 2022 with SUS data**	**2021 ONS Census [amended to 2001 grouping]**
England	1,708,430 (8.71)	1,955,095 (8.42)	4,995,225 (8.84)	583,770 (2.98)	659,410 (2.84)	2,381,720 (4.22)	367,980 (1.88)	466,220 (2.01)	1,669,380 (2.96)	16,468,610 (83.95)	19,566,635 (84.23)	45,783,400 (81.05)	489,350 (2.49)	581,395 (2.5)	1,660,315 (2.94)
East Midlands	319,445 (9.36)	360,305 (8.96)	368,130 (7.54)	97,320 (2.85)	110,695 (2.75)	129,985 (2.66)	60,985 (1.79)	81,860 (2.04)	117,245 (2.4)	2,867,445 (84.01)	3,386,935 (84.25)	4,179,775 (85.65)	67,880 (1.99)	80,350 (2)	84,915 (1.74)
East of England	300,605 (6.7)	342,735 (6.4)	367,425 (5.8)	147,865 (3.29)	168,770 (3.15)	184,950 (2.92)	89,825 (2)	116,180 (2.17)	179,655 (2.84)	3,856,555 (85.91)	4,617,355 (86.22)	5,478,365 (86.48)	94,055 (2.1)	110,425 (2.06)	124,675 (1.97)
London	368,555 (25.23)	403,495 (24.77)	1,670,120 (18.98)	123,080 (8.43)	138,875 (8.53)	1,188,370 (13.5)	60,995 (4.18)	70,810 (4.35)	505,775 (5.75)	775,090 (53.06)	853,735 (52.41)	4,731,170 (53.76)	133,035 (9.11)	162,100 (9.95)	704,290 (8)
North East	48,500 (5.31)	58,495 (5.3)	83,605 (3.16)	19,950 (2.18)	22,990 (2.08)	26,635 (1.01)	13,465 (1.47)	18,160 (1.65)	33,270 (1.26)	811,235 (88.75)	977,415 (88.57)	2,462,720 (93.04)	20,940 (2.29)	26,485 (2.4)	40,785 (1.54)
North West	68,150 (4.02)	78,725 (3.88)	568,635 (7.67)	17,470 (1.03)	19,705 (0.97)	173,920 (2.34)	14,420 (0.85)	19,055 (0.94)	163,245 (2.2)	1,570,265 (92.7)	1,882,895 (92.77)	6,347,395 (85.57)	23,535 (1.39)	29,350 (1.45)	164,205 (2.21)
South East	59,670 (4.71)	66,180 (4.4)	586,215 (6.32)	23,510 (1.86)	25,550 (1.7)	221,585 (2.39)	23,715 (1.87)	28,355 (1.89)	260,870 (2.81)	1,128,545 (89.15)	1,348,710 (89.74)	8,009,380 (86.33)	30,470 (2.41)	34,070 (2.27)	200,010 (2.16)
South West	71,795 (2.66)	78,600 (2.44)	132,670 (2.33)	25,735 (0.95)	28,730 (0.89)	69,615 (1.22)	33,950 (1.26)	42,945 (1.33)	114,075 (2)	2,529,835 (93.77)	3,026,520 (94)	5,309,610 (93.13)	36,640 (1.36)	42,795 (1.33)	75,220 (1.32)
West Midlands	174,370 (20.83)	194,405 (20.51)	760,965 (12.79)	65,400 (7.81)	72,195 (7.62)	269,020 (4.52)	29,220 (3.49)	33,420 (3.53)	178,225 (2.99)	540,185 (64.52)	615,885 (64.99)	4,585,025 (77.05)	28,055 (3.35)	31,815 (3.36)	157,525 (2.65)
Yorkshire and the Humber	294,930 (10.47)	369,235 (10.91)	457,465 (8.35)	62,195 (2.21)	70,370 (2.08)	117,645 (2.15)	40,435 (1.44)	54,080 (1.6)	117,015 (2.14)	2,365,245 (83.97)	2,828,245 (83.55)	4,679,965 (85.39)	54,135 (1.92)	63,245 (1.87)	108,685 (1.98)

Table 8: Count of patients with a recorded ethnicity in OpenSAFELY TPP [amended to the 2021 ethnicity grouping] (proportion of registered TPP population) and 2021 ONS Census counts (proportion of 2021 ONS Census population). All counts are rounded to the nearest 5.

	Asian			Black			Mixed			White			Other		
	**SNOMED 2022 (amended to 2021 grouping)**	**SNOMED 2022 with SUS data (amended to 2021 grouping)**	**2021 ONS Census**	**SNOMED 2022 (amended to 2021 grouping)**	**SNOMED 2022 with SUS data (amended to 2021 grouping)**	**2021 ONS Census**	**SNOMED 2022 (amended to 2021 grouping)**	**SNOMED 2022 with SUS data (amended to 2021 grouping)**	**2021 ONS Census**	**SNOMED 2022 (amended to 2021 grouping)**	**SNOMED 2022 with SUS data (amended to 2021 grouping)**	**2021 ONS Census**	**SNOMED 2022 (amended to 2021 grouping)**	**SNOMED 2022 with SUS data (amended to 2021 grouping)**	**2021 ONS Census**
England	1,885,400 (9.61)	2,141,950 (9.22)	5,426,390 (9.61)	583,770 (2.98)	659,460 (2.84)	2,381,720 (4.22)	367,985 (1.88)	466,360 (2.01)	1,669,380 (2.96)	16,468,610 (83.95)	19,566,465 (84.23)	45,783,400 (81.05)	312,375 (1.59)	394,495 (1.7)	1,229,150 (2.18)
East Midlands	344,275 (10.09)	386,490 (9.61)	391,105 (8.01)	97,320 (2.85)	110,675 (2.75)	129,985 (2.66)	60,990 (1.79)	81,900 (2.04)	117,245 (2.4)	2,867,440 (84.01)	3,386,870 (84.25)	4,179,775 (85.65)	43,050 (1.26)	54,200 (1.35)	61,945 (1.27)
East of England	339,585 (7.56)	384,345 (7.18)	405,870 (6.41)	147,865 (3.29)	168,785 (3.15)	184,950 (2.92)	89,830 (2)	116,190 (2.17)	179,655 (2.84)	3,856,550 (85.91)	4,617,335 (86.22)	5,478,365 (86.48)	55,075 (1.23)	68,805 (1.28)	86,230 (1.36)
London	411,250 (28.15)	447,805 (27.49)	1,817,640 (20.66)	123,085 (8.43)	138,915 (8.53)	1,188,370 (13.5)	60,995 (4.18)	70,835 (4.35)	505,775 (5.75)	775,090 (53.06)	853,765 (52.41)	4,731,170 (53.76)	90,345 (6.18)	117,735 (7.23)	556,770 (6.33)
North East	55,040 (6.02)	65,555 (5.94)	98,045 (3.7)	19,950 (2.18)	22,990 (2.08)	26,635 (1.01)	13,465 (1.47)	18,175 (1.65)	33,270 (1.26)	811,240 (88.75)	977,450 (88.57)	2,462,720 (93.04)	14,395 (1.57)	19,430 (1.76)	26,340 (1)
North West	77,785 (4.59)	89,135 (4.39)	622,685 (8.39)	17,470 (1.03)	19,715 (0.97)	173,920 (2.34)	14,425 (0.85)	19,065 (0.94)	163,245 (2.2)	1,570,260 (92.7)	1,882,875 (92.76)	6,347,395 (85.57)	13,900 (0.82)	18,975 (0.93)	110,155 (1.49)
South East	69,800 (5.51)	76,830 (5.11)	650,545 (7.01)	23,505 (1.86)	25,555 (1.7)	221,585 (2.39)	23,715 (1.87)	28,355 (1.89)	260,870 (2.81)	1,128,545 (89.15)	1,348,665 (89.74)	8,009,380 (86.33)	20,345 (1.61)	23,400 (1.56)	135,685 (1.46)
South West	86,810 (3.22)	94,535 (2.94)	159,185 (2.79)	25,735 (0.95)	28,725 (0.89)	69,615 (1.22)	33,955 (1.26)	42,970 (1.33)	114,075 (2)	2,529,835 (93.77)	3,026,510 (94)	5,309,610 (93.13)	21,635 (0.8)	26,855 (0.83)	48,705 (0.85)
West Midlands	185,085 (22.11)	205,620 (21.7)	794,265 (13.35)	65,400 (7.81)	72,190 (7.62)	269,020 (4.52)	29,225 (3.49)	33,435 (3.53)	178,225 (2.99)	540,185 (64.52)	615,820 (64.98)	4,585,025 (77.05)	17,345 (2.07)	20,605 (2.17)	124,225 (2.09)
Yorkshire and the Humber	313,230 (11.12)	388,560 (11.48)	487,055 (8.89)	62,195 (2.21)	70,380 (2.08)	117,645 (2.15)	40,435 (1.44)	54,070 (1.6)	117,015 (2.14)	2,365,245 (83.97)	2,828,215 (83.55)	4,679,965 (85.39)	35,835 (1.27)	43,900 (1.3)	79,095 (1.44)

Table 9: Count of individual ethnicity code use

Code	Term	Count
92491000000104	African - ethnic category 2001 census	369350
978231000000100	African: African, African Scottish or African British - Scotland ethnic category 2011 census	535
978251000000107	African: any other African - Scotland ethnic category 2011 census	195
413465009	Afro-Caribbean	1435
413466005	Afro-Caucasian	90
88971000000106	Albanian - ethnic category 2001 census	10245
94151000000105	Any other group - ethnic category 2001 census	44060
89001000000105	Arab - ethnic category 2001 census	53120
90027003	Arabs	2990
315280000	Asian - ethnic group	81595
92611000000106	Asian and Chinese - ethnic category 2001 census	5680
976831000000100	Asian or Asian British: Bangladeshi - England and Wales ethnic category 2011 census	4605
977731000000108	Asian or Asian British: Bangladeshi - Northern Ireland ethnic category 2011 census	600
976851000000107	Asian or Asian British: Chinese - England and Wales ethnic category 2011 census	5405
977751000000101	Asian or Asian British: Chinese - Northern Ireland ethnic category 2011 census	260
976791000000107	Asian or Asian British: Indian - England and Wales ethnic category 2011 census	32355
977591000000103	Asian or Asian British: Indian - Northern Ireland ethnic category 2011 census	5975
976811000000108	Asian or Asian British: Pakistani - England and Wales ethnic category 2011 census	17915
977711000000100	Asian or Asian British: Pakistani - Northern Ireland ethnic category 2011 census	2790
976871000000103	Asian or Asian British: any other Asian background - England and Wales ethnic category 2011 census	13365
977771000000105	Asian or Asian British: any other Asian background - Northern Ireland ethnic category 2011 census	1110
978171000000105	Asian or Asian Scottish or Asian British: Bangladeshi, Bangladeshi Scottish or Bangladeshi British - Scotland ethnic category 2011 census	185
978191000000109	Asian or Asian Scottish or Asian British: Chinese - Scotland ethnic category 2011 census	60
978111000000100	Asian or Asian Scottish or Asian British: Indian, Indian Scottish or Indian British - Scotland ethnic category 2011 census	1545
978071000000106	Asian or Asian Scottish or Asian British: Pakistani, Pakistani Scottish or Pakistani British - Scotland ethnic category 2011 census	1080
978211000000108	Asian or Asian Scottish or Asian British: any other Asian group - Scotland ethnic category 2011 census	310
88951000000102	Baltic States (Estonian or Latvian or Lithuanian) - ethnic category 2001 census	86330
186003008	Bangladeshi	29650

Table 9: Count of individual ethnicity code use

92471000000103	Bangladeshi or British Bangladeshi - ethnic category 2001 census	124530
315240009	Black - ethnic group	16180
185993005	Black - other African country	1425
185996002	Black - other Asian	820
185998001	Black - other, mixed	4230
18167009	Black African	135180
315635008	Black African and White	8365
275587000	Black Arab	455
185990008	Black British	17280
110791000000100	Black British - ethnic category 2001 census	31880
185988007	Black Caribbean	32445
315634007	Black Caribbean and White	12520
270460000	Black Caribbean/West India/Guyana	4155
275589002	Black East African Asian	185
270462008	Black East African Asian/Indo-Caribbean	255
309644006	Black Guyana	105
185995003	Black Indian sub-continent	310
275590006	Black Indo-Caribbean	55
275588005	Black Iranian	100
270461001	Black N African/Arab/Iranian	630
275586009	Black North African	270
309643000	Black West Indian	600
92581000000100	Black and Asian - ethnic category 2001 census	1210
92591000000103	Black and Chinese - ethnic category 2001 census	115
110771000000104	Black and White - ethnic category 2001 census	3000
976891000000104	Black or African or Caribbean or Black British: African - England and Wales ethnic category 2011 census	12425
977791000000109	Black or African or Caribbean or Black British: African - Northern Ireland ethnic category 2011 census	620
976911000000101	Black or African or Caribbean or Black British: Caribbean - England and Wales ethnic category 2011 census	5790
977811000000105	Black or African or Caribbean or Black British: Caribbean - Northern Ireland ethnic category 2011 census	315
976931000000109	Black or African or Caribbean or Black British: other Black or African or Caribbean background - England and Wales ethnic	3870

Table 9: Count of individual ethnicity code use

	category 2011 census	
977831000000102	Black or African or Caribbean or Black British: other Black or African or Caribbean background - Northern Ireland ethnic category 2011 census	600
185989004	Black, other, non-mixed origin	2750
93991000000103	Bosnian - ethnic category 2001 census	800
92681000000104	British Asian - ethnic category 2001 census	26100
186006000	British ethnic minority specified (NMO)	430
186007009	British ethnic minority unspecified (NMO)	665
92391000000108	British or mixed British - ethnic category 2001 census	9407055
29343004	Bulgarian	11685
718962008	Bulgarian Roma	115
107691000000105	Caribbean - ethnic category 2001 census	112475
92691000000102	Caribbean Asian - ethnic category 2001 census	535
270463003	Caribbean I./W.I./Guyana (NMO)	155
275591005	Caribbean Island (NMO)	280
978341000000102	Caribbean or Black: Black, Black Scottish or Black British - Scotland ethnic category 2011 census	110
978271000000103	Caribbean or Black: Caribbean, Caribbean Scottish or Caribbean British - Scotland ethnic category 2011 census	145
978361000000101	Caribbean or Black: any other Black or Caribbean group - Scotland ethnic category 2011 census	110
413773004	Caucasian (racial group)	12335
33897005	Chinese	48705
92511000000107	Chinese - ethnic category 2001 census	197525
92601000000109	Chinese and White - ethnic category 2001 census	2445
88961000000104	Commonwealth of (Russian) Independent States - ethnic category 2001 census	7325
186040000	Cook Island Maori	25
92571000000102	Cornish - ethnic category 2001 census	10390
94001000000108	Croatian - ethnic category 2001 census	1525
92791000000109	Cypriot (part not stated) - ethnic category 2001 census	1645
286009	Czech	7000
718959005	Czech Roma	115
270465005	E Afric Asian/Indo-Carib (NMO)	105
275596000	East African Asian (NMO)	295

Table 9: Count of individual ethnicity code use

92661000000108	East African Asian - ethnic category 2001 census	1840
110761000000106	English - ethnic category 2001 census	387530
69865008	Fijian	520
92771000000105	Filipino - ethnic category 2001 census	16010
275599007	Greek (NMO)	1570
93931000000104	Greek - ethnic category 2001 census	11685
275600005	Greek Cypriot (NMO)	400
93941000000108	Greek Cypriot - ethnic category 2001 census	2590
270466006	Greek/Greek Cypriot (NMO)	480
275593008	Guyana (NMO)	95
40182006	Gypsies	530
88931000000109	Gypsy/Romany - ethnic category 2001 census	12435
718963003	Hungarian Roma	3675
414481008	Indian (racial group)	203675
110751000000108	Indian or British Indian - ethnic category 2001 census	757205
186012005	Indian sub-continent (NMO)	825
275597009	Indo-Caribbean (NMO)	80
275595001	Iranian (NMO)	2565
89011000000107	Iranian - ethnic category 2001 census	19215
186014006	Irish (NMO)	705
92401000000106	Irish - ethnic category 2001 census	92830
977371000000109	Irish Traveller - Northern Ireland ethnic category 2011 census	10
88911000000101	Irish Traveller - ethnic category 2001 census	815
315283003	Irish traveller	420
94081000000103	Israeli - ethnic category 2001 census	535
93961000000109	Italian - ethnic category 2001 census	33230
414551003	Japanese	340
92761000000103	Japanese - ethnic category 2001 census	6925
92651000000105	Kashmiri - ethnic category 2001 census	1020
38361009	Koreans	1235

Table 9: Count of individual ethnicity code use

93981000000100	Kosovan - ethnic category 2001 census	1925
94091000000101	Kurdish - ethnic category 2001 census	13850
94111000000106	Latin American - ethnic category 2001 census	7125
92781000000107	Malaysian - ethnic category 2001 census	3760
94071000000100	Middle Eastern (excluding Israeli, Iranian and Arab) - ethnic category 2001 census	16600
92631000000103	Mixed Asian - ethnic category 2001 census	3735
92721000000106	Mixed Black - ethnic category 2001 census	2440
94021000000104	Mixed Irish and other White - ethnic category 2001 census	1525
315239007	Mixed ethnic census group	8585
976751000000104	Mixed multiple ethnic groups: White and Asian - England and Wales ethnic category 2011 census	4930
977431000000100	Mixed multiple ethnic groups: White and Asian - Northern Ireland ethnic category 2011 census	1535
976731000000106	Mixed multiple ethnic groups: White and Black African - England and Wales ethnic category 2011 census	2710
977411000000108	Mixed multiple ethnic groups: White and Black African - Northern Ireland ethnic category 2011 census	1140
976711000000103	Mixed multiple ethnic groups: White and Black Caribbean - England and Wales ethnic category 2011 census	5985
977391000000108	Mixed multiple ethnic groups: White and Black Caribbean - Northern Ireland ethnic category 2011 census	2065
976771000000108	Mixed multiple ethnic groups: any other Mixed or multiple ethnic background - England and Wales ethnic category 2011 census	5830
977551000000106	Mixed multiple ethnic groups: any other Mixed or multiple ethnic background - Northern Ireland ethnic category 2011 census	325
978051000000102	Mixed or multiple ethnic groups: any Mixed or multiple ethnic group - Scotland ethnic category 2011 census	65
414752008	Mixed racial group (racial group)	2910
94101000000109	Moroccan - ethnic category 2001 census	3975
94121000000100	Multi-ethnic islands: Mauritian or Seychellois or Maldivian or St Helena - ethnic category 2001 census	2305
270464009	N African Arab/Iranian (NMO)	1700
718131000000106	Nepali	5570
186036009	New Zealand European	505
186039002	New Zealand Maori	75
186035008	New Zealand ethnic groups	560
92731000000108	Nigerian - ethnic category 2001 census	11965
94061000000107	North African - ethnic category 2001 census	5180
275594002	North African Arab (NMO)	1690
92561000000109	Northern Irish - ethnic category 2001 census	935

Table 9: Count of individual ethnicity code use

414978006	Oriental	170
92521000000101	Other - ethnic category 2001 census	241880
186010002	Other African countries (NMO)	1310
186013000	Other Asian (NMO)	6430
92481000000101	Other Asian background - ethnic category 2001 census	305220
315281001	Other Asian ethnic group	57510
92701000000102	Other Asian or Asian unspecified - ethnic category 2001 census	15905
186000006	Other Black - Black/Asian orig	625
185999009	Other Black - Black/White orig	1870
92501000000105	Other Black background - ethnic category 2001 census	52135
92741000000104	Other Black or Black unspecified - ethnic category 2001 census	5080
186017004	Other European (NMO)	33265
186037000	Other European in New Zealand	50
92451000000107	Other Mixed background - ethnic category 2001 census	122360
92621000000100	Other Mixed or Mixed unspecified - ethnic category 2001 census	7075
94041000000106	Other White European or European unspecified or Mixed European - ethnic category 2001 census	160190
92411000000108	Other White background - ethnic category 2001 census	1431975
94051000000109	Other White or White unspecified - ethnic category 2001 census	57390
315279003	Other black ethnic group	8495
976951000000102	Other ethnic group: Arab - England and Wales ethnic category 2011 census	4015
977851000000109	Other ethnic group: Arab - Northern Ireland ethnic category 2011 census	150
978381000000105	Other ethnic group: Arab, Arab Scottish or Arab British - Scotland ethnic category 2011 census	280
976971000000106	Other ethnic group: any other ethnic group - England and Wales ethnic category 2011 census	12815
977871000000100	Other ethnic group: any other ethnic group - Northern Ireland ethnic category 2011 census	170
978401000000105	Other ethnic group: any other ethnic group - Scotland ethnic category 2011 census	75
186005001	Other ethnic non-mixed (NMO)	14085
186021006	Other ethnic, Asian/White origin	11335
186020007	Other ethnic, Black/White origin	1060
186019001	Other ethnic, mixed origin	14140
186022004	Other ethnic, mixed white origin	3060

Table 9: Count of individual ethnicity code use

186023009	Other ethnic, other mixed origin	4105
94031000000102	Other mixed White - ethnic category 2001 census	11610
94011000000105	Other republics which made up the former Yugoslavia - ethnic category 2001 census	1625
401214002	Other white British ethnic group	20850
186002003	Pakistani	103375
92461000000105	Pakistani or British Pakistani - ethnic category 2001 census	540820
88941000000100	Polish - ethnic category 2001 census	215080
718964009	Polish Roma	455
80208004	Portuguese	12365
92641000000107	Punjabi - ethnic category 2001 census	5350
160531006	Race: West indian	80
718958002	Roma	580
445343003	Romanian	86070
718960000	Romanian Roma	975
86275006	Samoan	55
92541000000108	Scottish - ethnic category 2001 census	7655
88981000000108	Serbian - ethnic category 2001 census	1365
110781000000102	Sinhalese - ethnic category 2001 census	310
36329002	Slovak	11000
718961001	Slovak Roma	2500
92711000000100	Somali - ethnic category 2001 census	17665
186044009	South East Asian	1190
89021000000101	South and Central American - ethnic category 2001 census	9300
86461000000107	Sri Lankan - ethnic category 2001 census	27370
92671000000101	Tamil - ethnic category 2001 census	2665
81560001	Tongan	30
88921000000107	Traveller - ethnic category 2001 census	26330
275601009	Turkish (NMO)	2415
110401000000103	Turkish - ethnic category 2001 census	16270
275602002	Turkish Cypriot (NMO)	215

Table 9: Count of individual ethnicity code use

93951000000106	Turkish Cypriot - ethnic category 2001 census	1415
270467002	Turkish/Turkish Cypriot (NMO)	970
93921000000101	Ulster Scots - ethnic category 2001 census	20
312859007	Vietnamese	2145
92751000000101	Vietnamese - ethnic category 2001 census	4450
92551000000106	Welsh - ethnic category 2001 census	4510
275592003	West Indian (NMO)	360
977351000000100	White - Northern Ireland ethnic category 2011 census	200
185984009	White - ethnic group	920270
315236000	White British	5746445
494131000000105	White British - ethnic category 2001 census	4156730
315237009	White Irish	37325
494161000000100	White Irish - ethnic category 2001 census	25740
401213008	White Scottish	7505
92441000000109	White and Asian - ethnic category 2001 census	92835
92431000000100	White and Black African - ethnic category 2001 census	89460
92421000000102	White and Black Caribbean - ethnic category 2001 census	93915
976631000000101	White: English or Welsh or Scottish or Northern Irish or British - England and Wales ethnic category 2011 census	726825
976671000000104	White: Gypsy or Irish Traveller - England and Wales ethnic category 2011 census	735
977971000000108	White: Gypsy or Irish Traveller - Scotland ethnic category 2011 census	25
976651000000108	White: Irish - England and Wales ethnic category 2011 census	7315
977951000000104	White: Irish - Scotland ethnic category 2011 census	70
978011000000101	White: Polish - Scotland ethnic category 2011 census	2595
977911000000103	White: Scottish - Scotland ethnic category 2011 census	500
976691000000100	White: any other White background - England and Wales ethnic category 2011 census	71540
978031000000109	White: any other White ethnic group - Scotland ethnic category 2011 census	1290
977931000000106	White: other British - Scotland ethnic category 2011 census	2220
296841000000102	Yemeni	590
